# Anatomic healing after non-operative treatment of a large, displaced anterior glenoid rim fracture after primary traumatic anterior shoulder dislocation – a case report

**DOI:** 10.1186/s12891-020-03384-1

**Published:** 2020-06-09

**Authors:** Lukas Ernstbrunner, Malik Jessen, Karl Wieser

**Affiliations:** Department of Orthopedics, Balgrist University Hospital, University of Zurich, Forchstrasse 340, 8008 Zurich, Switzerland

**Keywords:** Anterior shoulder dislocation, Primary dislocation, Anterior glenoid rim, Fracture, Non-operative management

## Abstract

**Background:**

Large, displaced anterior glenoid rim fractures after primary traumatic anterior shoulder dislocation are usually managed by surgical stabilization. Although there is little evidence supporting surgical management, it is often preferred over non-operative treatment. This case report describes non-operative management of such large, displaced anterior glenoid rim fracture with CT- and MRI-based documentation of anatomical healing of the fracture fragment, a finding that has not been described previously.

**Case presentation:**

This case report describes a 49-year-old male, right-hand dominant, carpenter, who had a left-sided primary anterior shoulder dislocation after a fall while skiing. Initial plain radiographs showed a reduced glenohumeral joint with a large, displaced anterior glenoid rim fracture. CT-evaluation showed a centered humeral head, and as per our institutional protocol, non-operative management was initiated. Longitudinal radiographic assessment at 2 weeks, 4.5 months and 12 months showed reduction of the initially severely displaced fracture fragment. MRI- and CT-evaluation after 12 months confirmed anatomical healing of the fragment. At final follow-up, the patient was highly satisfied, although the healing process was complicated by posttraumatic frozen shoulder, which has had almost fully resolved after 12 months.

**Conclusions:**

Given that the glenohumeral joint is concentrically reduced, large (displaced) anterior glenoid rim fractures after traumatic primary shoulder dislocation can be successfully treated non-operatively, with the potential of anatomical fracture fragment healing. Therefore, it remains subject to conservative treatment at our institution and surgical stabilization is reserved for patients with a decentered humeral head or persistent glenohumeral instability.

## Background

Of all scapular fractures, around 10% are glenoid rim fractures [1–3]. Anterior glenoid rim fractures are strongly associated with primary traumatic shoulder dislocation [4, 5] and based on Ideberg et al. [1], classified as Type Ia if the fracture fragment is < 5 mm, and as Type Ib if the fracture fragment is > 5 mm.

There is still a controversy about the management of large anterior glenoid rim fractures. Although there is little evidence supporting surgical management, it is often preferred with the argument of anatomical reduction, and therefore theoretically reduced risk of redislocation and posttraumatic osteoarthritis (OA). Surgical management is generally recommended when anterior glenoid rim fractures are associated with a decentered humeral head [6], fracture fragment > 5 mm (i.e. Ideberg Type Ib) [7, 8] or fragment displacement greater than 5 mm [9, 10].

Non-operative treatment is accepted in anterior glenoid rim fractures with small, so-called chip fractures [11] or Ideberg Type Ia fractures, respectively. However, non-operative treatment may also be considered in situations with a large fracture fragment. One of the few available studies was published by the reporting institution and the results 6 years after conservative treatment in 14 consecutive patients with large, displaced anterior glenoid rim fractures showed a stable, pain free and functional shoulder in all patients, without any reported complications, redislocations, conducted or planned surgical interventions [6]. CT-evaluation revealed a mean step-off of 3.0 mm, however, only 3 out of 14 patients showed mild to moderate (clinically asymptomatic) OA, all of which with a tendency towards anterior humeral head subluxation. Ever since, large anterior glenoid rim fractures with a centered humeral head are treated non-operatively at our institution.

To the best of our knowledge, there is no (CT- and MRI-based) documentation of anatomical healing of large, displaced anterior glenoid rim fractures. This case report describes such successful anatomic healing of a non-operatively managed large, displaced anterior glenoid rim fracture after primary traumatic anterior shoulder dislocation in a 49-year-old male patient.

## Case presentation

A 49-year-old male, right-hand dominant, carpenter had a left-sided primary anterior shoulder dislocation after a fall while skiing. He had no previous shoulder pathology. The shoulder was reduced in an outside trauma practice, immobilized in internal rotation in a sling and the patient was referred to our shoulder department.

**Fig. 1:**
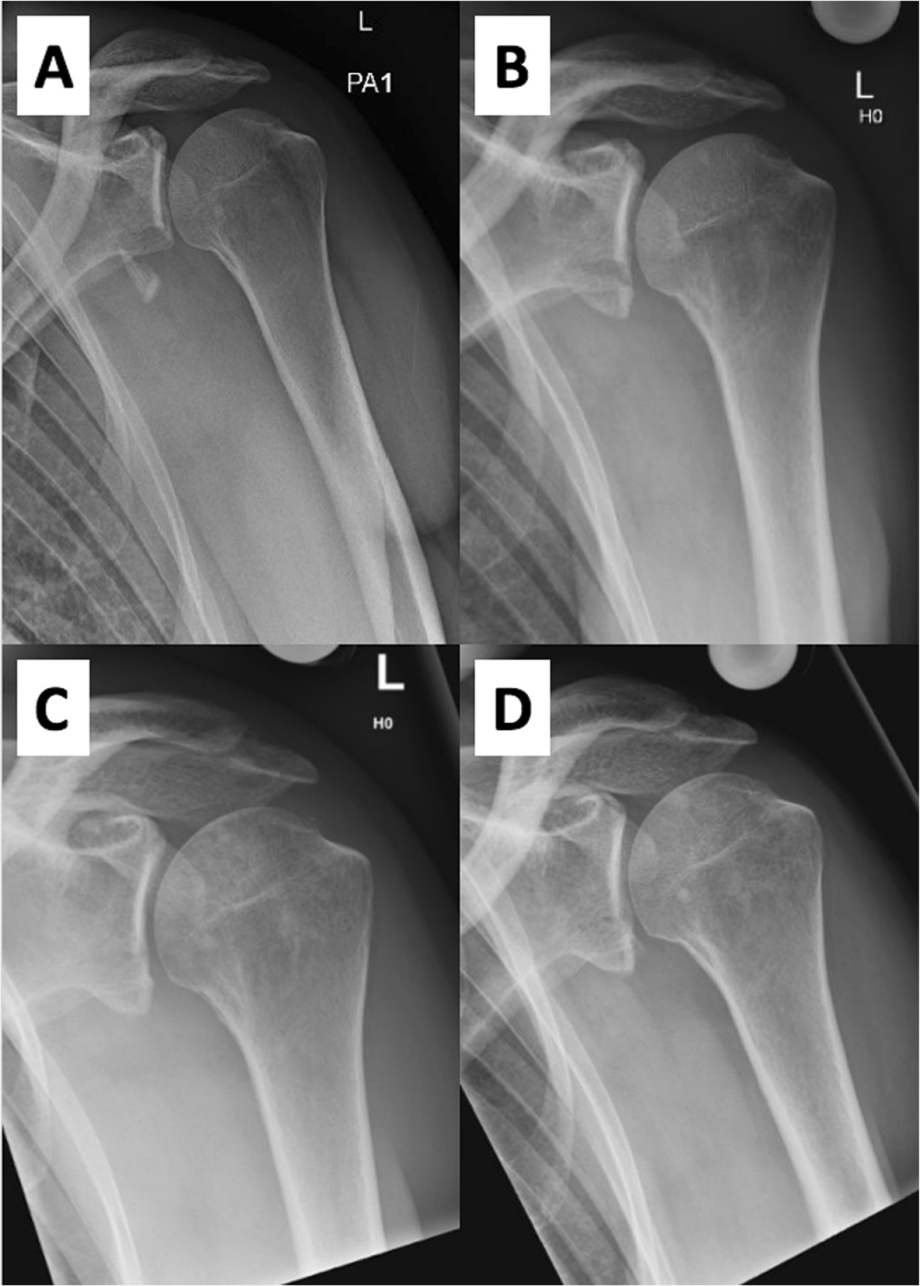
**a** to **d** Series of plain ap radiographs over 12 months after trauma. **a** Initial radiograph showing a large, displaced anterior glenoid rim fracture. **b** 2 weeks after trauma, realignment of the fracture fragment is visible. **c** Radiograph 4.5 months after trauma shows consolidation of the fragment. **d** 12 months after trauma, the large fracture fragment is healed

At initial presentation 4 days after trauma, clinical examination showed good external and internal rotation strength, no external rotation lag and a negative belly-press test. He had almost full range of motion (ROM), anterior apprehension and abduction strength were not assessable due to pain. The patient had also no axillary nerve symptoms. Plain ap and true lateral radiographs showed a reduced glenohumeral joint with a large, displaced anterior glenoid rim fracture (Fig. 1a).

**Fig. 2:**
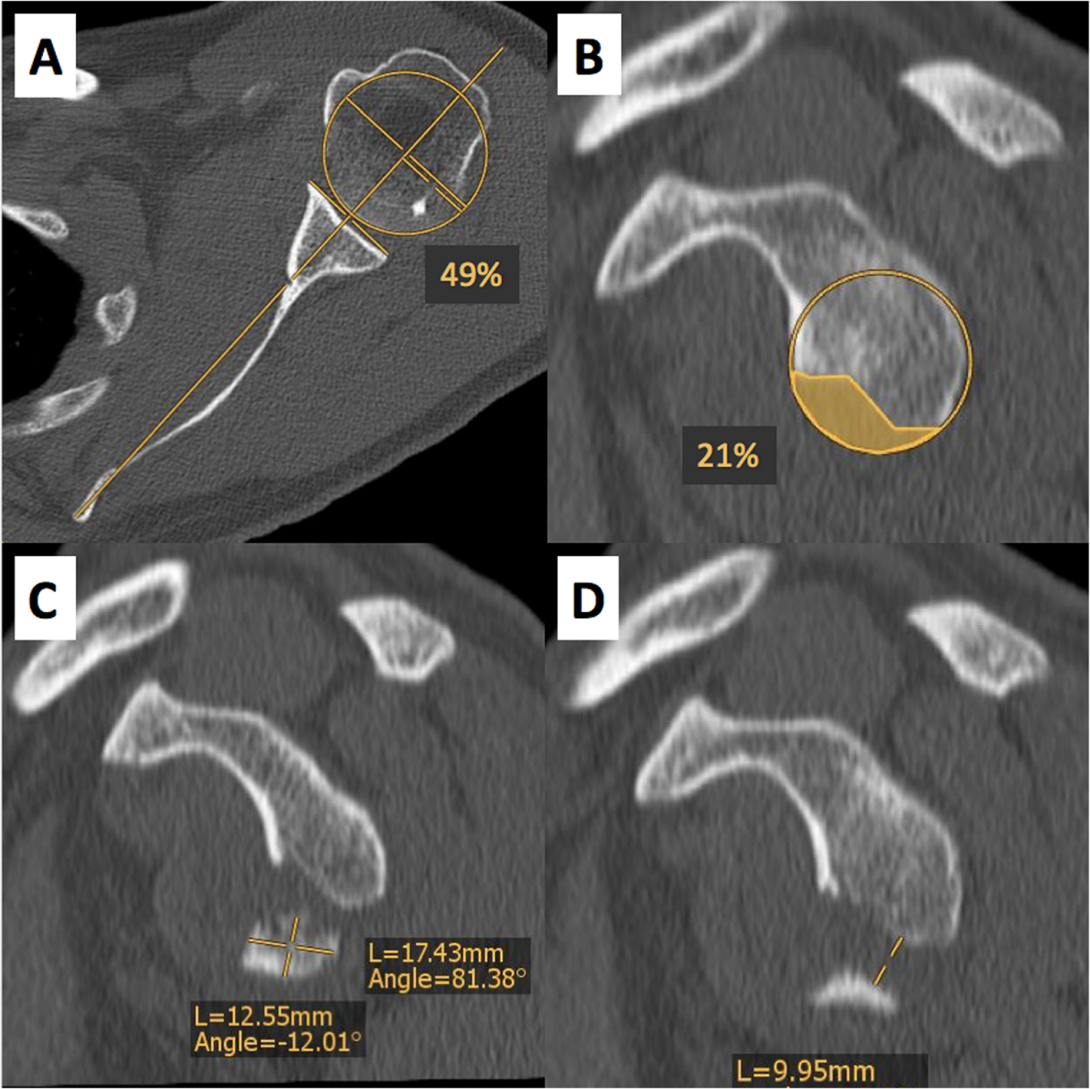
**a** to **d** CT-based assessment of humeral head centering, glenoid bone loss, fracture fragment size and displacement. **a** Axial CT-scan shows a centered humeral head of 49% (normal range; 35–65%). **b** Anterior glenoid bone loss in the en face view measures 21%. **c** The dimensions of the fracture fragment in the en face view are 17 mm × 13 mm. **d** Fracture fragment displacement in the en face view is 10 mm

CT-scan evaluation revealed a centered humeral head (49%) according to Gerber et al. [12], anterior glenoid bone loss in the en face view of 21% (based on the pico method [13–15]), a fracture fragment of 17 mm × 13 mm (i.e. Ideberg Type Ib [1]) and fragment displacement of 10 mm (Fig. 2).

Non-operative management was continued with physiotherapy twice a week, daily pendulum exercises, and active elevation without combined abduction and external rotation and weight bearing for 6 weeks. Sling immobilization in internal rotation was recommended for 2 weeks.

The patient was rescheduled 2 weeks later for another plain radiographs and clinical examination. He reported no symptoms of instability with slightly impaired active and passive ROM. Radiographs showed realignment of the fracture fragment (Fig. 1b) and a centered humeral head. At 6 weeks after trauma, the patient had developed symptoms of posttraumatic stiffness with passive external rotation with the arm at the side limited to 0° (50° contralateral side) and passive glenohumeral abduction at 70° (90° contralateral side). Conservative treatment was adapted with daily NSAIDs and pain-free physiotherapy for another 6 weeks.

**Fig. 3:**
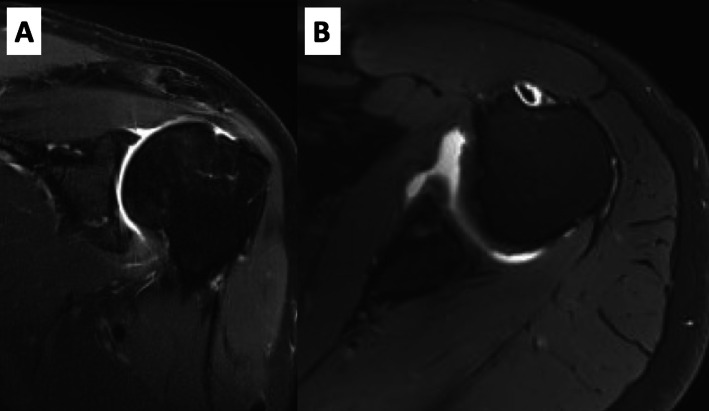
**a** and **b** MR-imaging 4.5 months after trauma. **a** Coronal MR-imaging shows an obliterated axillary recess. The fracture fragment is aligned with the glenoid. **b** Axial MR-imaging shows an anatomically aligned fracture fragment without step-formation

At 4.5 months, the patient had still a stable shoulder, was able to return to work for 100%, but was stiff (passive ROM: glenohumeral abduction 40°; external rotation 10°) and slightly painful. Radiographs (Fig. 1c) and MR-imaging were performed, which showed no rotator cuff pathology but an obliterated axillary recess, confirming the posttraumatic frozen shoulder. The articular surface of the glenoid showed an anatomic realignment of the fracture fragment (Fig. 3).

**Fig. 4:**
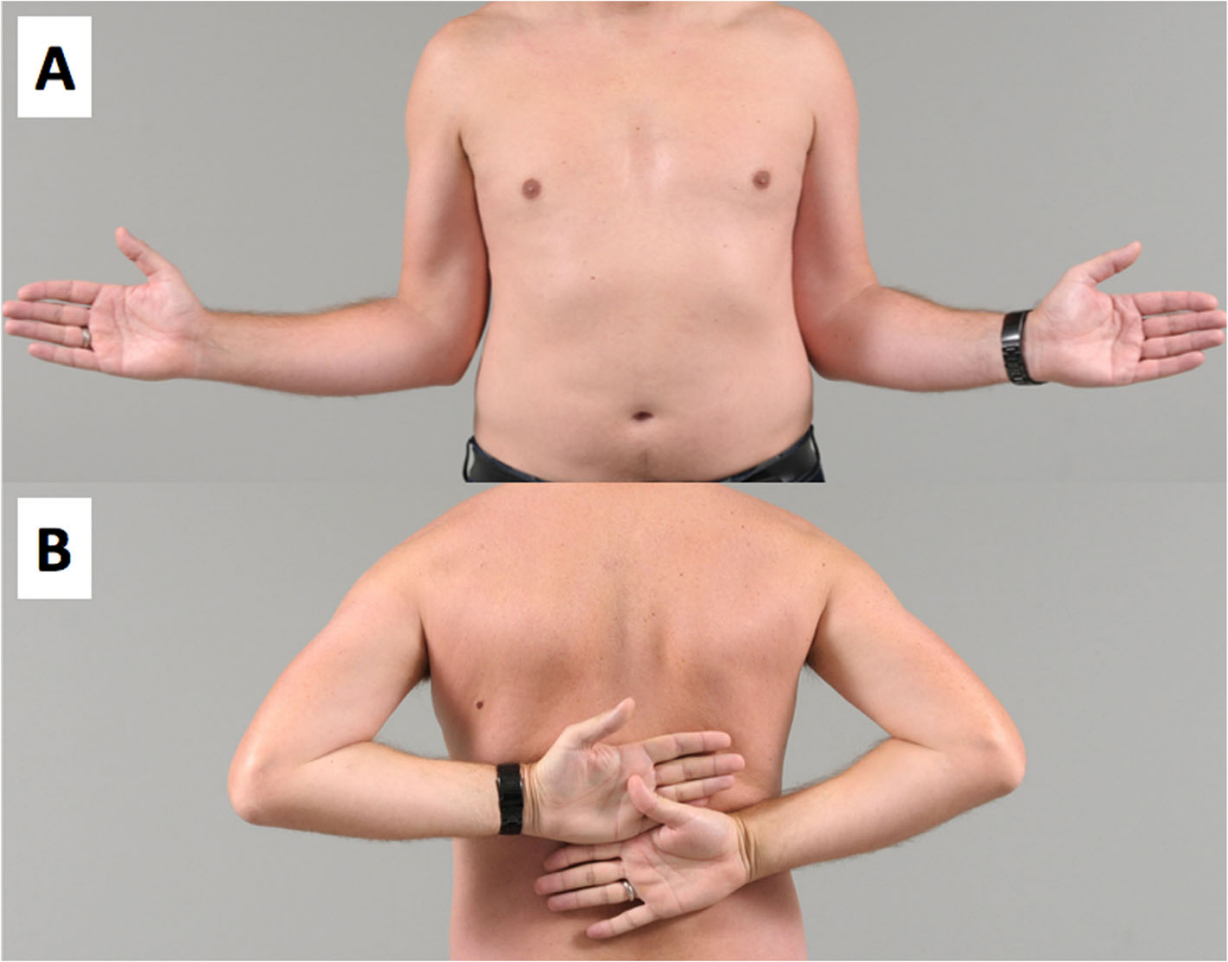
**a** and **b** Clinical photographs made 12 months after trauma of the left shoulder. **a** Active external rotation with − 10° on the left compared with the healthy, contralateral side, and (**b**) free active internal rotation

At 12 months after trauma, the patient was painfree, had a stable shoulder and negative apprehension test, and active as well as passive ROM was almost free (passive ROM: glenohumeral abduction symmetrical 90°; external rotation 40° (50° contralateral side)) with normal shoulder strength (Fig. 4). The patient was highly satisfied, had a subjective shoulder value [16] of 100%, a relative Constant Murley Score [17] of 91% and a Western Ontario Shoulder Instability index of 97%.

**Fig. 5:**
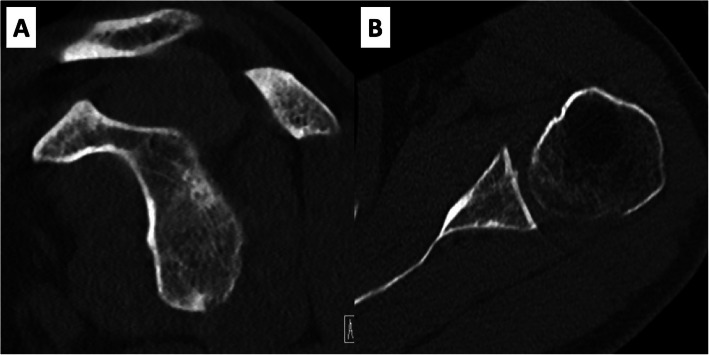
**a** and **b** CT-scans 12 months after trauma of the left shoulder. **a** The glenoid in the en face view shows healing of the large fracture fragment. **b** The axial plane shows a step-free and anatomic healing of the fragment with a centered humeral head (52%)

Radiographs showed anatomical alignment of the fracture fragment (Fig. 1d) and non-routine CT-scans confirmed healing of the fragment and a centered humeral head (Fig. 5).

## Discussion and conclusions

Our case report shows that even large, displaced anterior glenoid rim fractures after primary shoulder dislocation can be successfully treated with non-operative management and can heal in an anatomical position, a finding that has not been described previously. Besides (nearly) fully restored shoulder function and high patient satisfaction, MRI- and CT-evaluation revealed anatomic healing of the fracture fragment and centered humeral head 12 months after trauma.

The good functional outcome in this case report is in accordance with the previously published results of our institution [6]. In 14 consecutive patients with large anterior glenoid rim fractures no redislocation was reported after conservative treatment. Also, there were no remaining functional disabilities after a mean of 6 six years. On the other side, the results of arthroscopic techniques showed significant functional impairment. Both, Porcellini et al. [18] and Scheibel et al. [7] reported a mean loss of external rotation of 10° compared to the healthy contralateral side. Further (rare) complications of surgical stabilization of anterior glenoid rim fractures are nerve palsy, chronic pain, infection, or the early onset of (posttraumatic or even iatrogenic) OA [7, 19–22]. In contrast to such permanent postoperative impairments, the pathophysiology of posttraumatic frozen shoulder is different and usually resolves anywhere between 12 and 42 months [23].

The longitudinal radiographic follow-up in our case showed a reduction of the severely displaced fracture fragment. Within the first posttraumatic weeks, the fragment realigned anatomically, which could be the result of capsule shrinkage after traumatic capsular avulsion and the probability that large fragments remain in continuity with the capsuloligamentous complex. The drawback in our patient was the development of a posttraumatic stiffness, which, however, could be treated conservatively with only minor functional deficits after 12 months, which usually resolves further along [23].

In the study of Maquieira et al. [6], bony healing was obtained in all cases and similar results were observed by Kraus et al. [24]. Even if the fragment in the cohort of Maquieira et al. healed in all patients with a mean step-off of 3.0 mm, only 3 out of 14 patients showed mild or moderate OA, all of which were clinically asymptomatic. Anatomic reduction is thought to be more reliable with surgical stabilization. However, radiographic results after arthroscopic reconstruction showed also in the biggest published cohort in 7 out of 21 patients a postoperative step-off, which was in 4 of these 7 patients not associated with radiographic signs of OA, nor was there a statistical correlation between a step-off and postoperative OA. On the other side, 6 out of 21 patients showed radiographic signs of OA after arthroscopic stabilization, and 3 of those were graded to have severe OA [7]. It seems that an intraarticular step-off is well tolerated, but surgical stabilization of anterior glenoid rim fracture may accelerate the process of degeneration.

Another factor affecting outcome is the position of the humeral head. The 3 out of 14 patients in our previous series with mild to moderate OA had a tendency toward anterior humeral head subluxation [6]. The humeral head in our patient was centered on the initial CT-scan as well as after 12 months. Therefore, the success of conservative management depends on the position of the humeral head, which should be centered after shoulder reduction.

Given that the glenohumeral joint is concentrically reduced, large (displaced) anterior glenoid rim fractures after primary traumatic shoulder dislocation can be successfully treated non-operatively, with the potential of anatomical fracture fragment healing. Therefore, it remains subject to conservative treatment at our institution and surgical stabilization is reserved for patients with a decentered humeral head or persistent glenohumeral instability.

## Data Availability

All data and materials are included in the manuscript and thus available to the reader.

## Abbreviations

OA: osteoarthritis
CT: computed tomography
MRI: magnetic resonance imaging
ROM: range of motion

## References

[1] Ideberg R, Grevsten S, Larsson S (1995). Epidemiology of scapular fractures. Incidence and classification of 338 fractures. Acta Orthop Scand.

[2] Tucek M, Chochola A, Klika D, Bartonicek J (2017). Epidemiology of scapular fractures. Acta Orthop Belg.

[3] Goss TP (1992). Fractures of the glenoid cavity. J Bone Joint Surg Am.

[4] Bigliani LU, Newton PM, Steinmann SP, Connor PM, McLlveen SJ (1998). Glenoid rim lesions associated with recurrent anterior dislocation of the shoulder. Am J Sports Med.

[5] Schandelmaier P, Blauth M, Schneider C, Krettek C (2002). Fractures of the glenoid treated by operation. A 5- to 23-year follow-up of 22 cases. J Bone Joint Surg (Br).

[6] Maquieira GJ, Espinosa N, Gerber C, Eid K (2007). Non-operative treatment of large anterior glenoid rim fractures after traumatic anterior dislocation of the shoulder. J Bone Joint Surg British Vol.

[7] Scheibel M, Hug K, Gerhardt C, Krueger D (2016). Arthroscopic reduction and fixation of large solitary and multifragmented anterior glenoid rim fractures. J Shoulder Elb Surg.

[8] Kummel BM (1970). Fractures of the glenoid cusing chronic dislocation of the shoulder. Clin Orthop Relat Res.

[9] van Oostveen DP, Temmerman OP, Burger BJ, van Noort A, Robinson M (2014). Glenoid fractures: a review of pathology, classification, treatment and results. Acta Orthop Belg.

[10] De Palma AF (1983). Fractures and fracture-dislocations of the shoulder girdle.

[11] Hovelius L, Eriksson K, Fredin H, Hagberg G, Hussenius A, Lind B (1983). Recurrences after initial dislocation of the shoulder. Results of a prospective study of treatment. J Bone Joint Surg Am.

[12] Gerber C, Costouros JG, Sukthankar A, Fucentese SF (2009). Static posterior humeral head subluxation and total shoulder arthroplasty. J Shoulder Elb Surg.

[13] Ernstbrunner L, Plachel F, Heuberer P, Pauzenberger L, Moroder P, Resch H (2018). Arthroscopic versus open iliac crest bone grafting in recurrent anterior shoulder instability with glenoid bone loss: a computed tomography-based quantitative assessment. Arthroscopy.

[14] Ernstbrunner L, Wartmann L, Zimmermann SM, Schenk P, Gerber C, Wieser K (2019). Long-term results of the open Latarjet procedure for recurrent anterior shoulder instability in patients older than 40 years. Am J Sports Med.

[15] Magarelli N, Milano G, Sergio P, Santagada DA, Fabbriciani C, Bonomo L (2009). Intra-observer and interobserver reliability of the Pico computed tomography method for quantification of glenoid bone defect in anterior shoulder instability. Skelet Radiol.

[16] Gilbart MK, Gerber C (2007). Comparison of the subjective shoulder value and the Constant score. J Shoulder Elb Surg.

[17] Constant CR, Murley AH (1987). A clinical method of functional assessment of the shoulder. Clin Orthop Relat Res.

[18] Porcellini G, Campi F, Paladini P (2002). Arthroscopic approach to acute bony Bankart lesion. Arthroscopy.

[19] Mayo KA, Benirschke SK, Mast JW (1998). Displaced fractures of the glenoid fossa. Results of open reduction and internal fixation. Clin Orthop Relat Res.

[20] Scheibel M, Magosch P, Lichtenberg S, Habermeyer P (2004). Open reconstruction of anterior glenoid rim fractures. Knee Surg Sports Traumatol Arthrosc.

[21] Seybold D, Gekle C, Muhr G, Kalicke T (2006). Severe complications after percutaneous transaxillary refixation of a glenoid rim fracture. Unfallchirurg.

[22] Tauber M, Moursy M, Eppel M, Koller H, Resch H (2008). Arthroscopic screw fixation of large anterior glenoid fractures. Knee Surg Sports Traumatol Arthrosc.

[23] Harryman DT, Lazurus MD, Rozencwaig R, Rockwood CA, Matsen F, Wirth MA, Lippitt SB (2004). The stiff shoulder. The shoulder.

[24] Kraus N, Gerhardt C, Haas N, Scheibel M (2010). Conservative therapy of antero-inferior glenoid fractures. Unfallchirurg.

